# Beta oscillations following performance feedback predict subsequent recall of task-relevant information

**DOI:** 10.1038/s41598-020-72128-x

**Published:** 2020-09-15

**Authors:** Azadeh HajiHosseini, Cendri A. Hutcherson, Clay B. Holroyd

**Affiliations:** 1 Department of Psychology, University of Victoria, P. O. Box 1700 STN CSC, Victoria, BC V8W 2Y2 Canada; 2 Department of Psychology, University of Toronto Scarborough, 1265 Military Trail, Toronto, ON M1C 1A4 Canada; 3 Department of Marketing, Rotman School of Management, University of Toronto, 105 St George Street, Toronto, ON M5S 3E6 Canada; 4 Department of Experimental Psychology, Faculty of Psychology and Educational Sciences, Ghent University, Henri Dunantlaan 2, 9000 Ghent, Belgium; 5 Present Address: Department of Psychology, University of Toronto Scarborough, 1265 Military Trail, Toronto, ON M1C 1A4 Canada

**Keywords:** Cognitive neuroscience, Cognitive control, Motivation

## Abstract

Reward delivery in reinforcement learning tasks elicits increased beta power in the human EEG over frontal areas of the scalp but it is unclear whether these 20–30 Hz oscillations directly facilitate reward learning. We previously proposed that frontal beta is not specific to reward processing but rather reflects the role of prefrontal cortex in maintaining and transferring task-related information to other brain areas. To test this proposal, we had subjects perform a reinforcement learning task followed by a memory recall task in which subjects were asked to recall stimuli associated either with reward feedback (Reward Recall condition) or error feedback (Error Recall condition). We trained a classifier on post-feedback beta power in the Reward Recall condition to discriminate trials associated with reward feedback from those associated with error feedback and then tested the classifier on post-feedback beta power in the Error Recall condition. Crucially, the model classified error-related beta in the Error Recall condition as reward-related. The model also predicted stimulus recall from post-feedback beta power irrespective of feedback valence and task condition. These results indicate that post-feedback beta power is not specific to reward processing but rather reflects a more general task-related process.

## Introduction

Neural oscillations associated with prefrontal cortex (PFC) function have been proposed to reflect neurocognitive processes related to learning, working memory, and cognitive control^1–4^. Several theoretical accounts hold that these control processes are governed by principles of reinforcement learning^5–7^. Consistent with this possibility, electroencephalogram (EEG) studies have revealed an increase in beta oscillatory power over frontal areas of the scalp that peaks around 350 ms following the delivery of reward-related feedback stimuli in a variety of reinforcement learning tasks^8–11^. These findings have raised the question of whether beta oscillations have an active role in reinforcement learning^12^ by indexing a signal that is specific to rewards. For example, some researchers have suggested that learning from feedback is driven by an oscillatory mechanism that depends on the valence of the eliciting feedback; on this view, beta oscillations facilitate learning from reward feedback by synchronizing the activity of multiple brain areas such as the dorsolateral PFC (DLPFC), frontopolar cortex, and motor cortex^13^. A different account suggests that frontal beta oscillations govern the execution of cognitive processes related to motivation, attention, and memory that are specifically elicited by positive performance feedback^14^. However, these proposals await empirical verification.

By contrast to the above accounts, we have recently proposed that frontal beta is not related to reward per se, but rather is related to adaptive task performance based on relevant information received from feedback, irrespective of its valence. According to this proposal, DLPFC-mediated beta reflects a working memory process involved in the activation of neural ensembles that encode task-related information^15^. On this view, frontal beta power is not specifically related to rewards but rather is elicited by feedback stimuli in accord with their task relevance. The task-related information is then communicated to other brain areas responsible for task execution, including posterior cortical areas and the hippocampus, in order to facilitate behavioral adaptation.

Here we tested this hypothesis by experimentally manipulating the task-relevance of reward feedback using a dual-task paradigm. In a “No Recall” (NR) condition, which served as a control, participants engaged in a relatively standard reinforcement learning task where subjects selected decks of cards over multiple trials with the goal of making money. We predicted that beta power in this condition would track reward feedback rather than error feedback as observed previously. Further, in a “Reward Recall” (RR) condition participants were required to do the same task as in the NR condition while also remembering the choices that led to reward feedback, to be recalled later at the end of each block of trials. We predicted that because the demands of the working memory task were congruent with the demands of the reinforcement learning task in this condition, beta power would similarly track reward feedback rather than error feedback. Crucially, in an “Error Recall” (ER) condition, participants were instructed to remember the choices that led to *error* feedback and to recall those choices at the end of each block of trials. We predicted that these task demands would reverse the association between outcomes and beta oscillations such that beta power would track error feedback more than reward feedback.

We verified these predictions using a classification approach based on linear discriminant analysis. In contrast to more standard approaches that are based on inferential statistics, this approach provided a means to test how similarly post-feedback beta power over frontal and posterior brain areas behaved on trials in which the stimuli were rewarded with trials in which the stimuli were to be remembered. In other words, this approach enabled us to assess the similarity of beta power across the different conditions. By contrast, application of null hypothesis significance tests would indicate whether conditions are statistically different from each other rather than their similarity. In this way, we sought to verify whether post-feedback beta power over frontal and posterior brain areas is sensitive to task-relevance as opposed to rewards per se.

## Materials and methods

### Participants

Thirty undergraduate students (9 male, 20.0 ± 1.6 years old) at the University of Victoria participated in the experiment. Subjects acquired extra course credits for their participation and were also paid a monetary bonus for their performance. The study was conducted in accordance with the ethical standards prescribed in the Declaration of Helsinki and was approved by the human subjects review board at the University of Victoria. Informed written consent was obtained from participants prior to the experiment.

### Paradigm

#### Task

**Figure 1:**
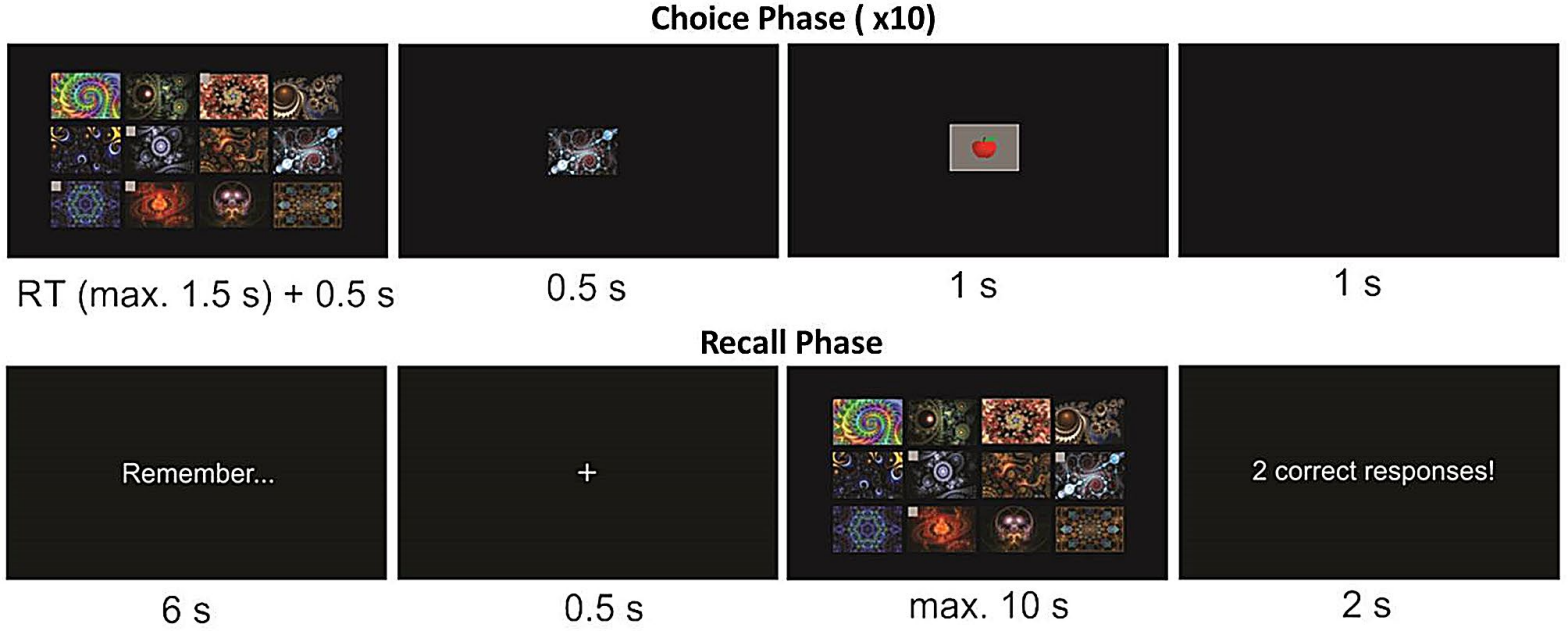
Task timeline. A choice phase on each block consisted of 10 trials where on each trial subjects selected a deck of cards. Twelve abstract stimuli representing decks were presented at the same locations on each trial throughout the block. Each deck could be selected only once in 10 trials; on each trial the previously selected decks on that block were marked with a small gray square in the corner of the image (top row, far left). In the RR and ER conditions only, a recall phase occurred after the completion of each block of 10 trials. Participants were given 6 s to recall the previous sequence of choices and outcomes on that block, and were then, following the brief presentation of a fixation stimulus, shown the 12 stimuli in the same locations. They were instructed to select the target decks (reward in the RR and error in the ER conditions); upon selecting all of the targets or after 10 s elapsed, whichever came first, the number of correct recall responses were presented on the screen.

We used a paradigm wherein we could manipulate the relevance of reward and error feedback for optimal task performance. This paradigm consisted of a reinforcement learning task presented to subjects as a card choice task that they played for monetary reward, followed by a recall task. In the No Recall (NR) condition, subjects only performed the reinforcement learning task. In the Reward Recall (RR) condition, we asked them to remember the stimuli associated with reward feedback for subsequent recall. In the Error Recall (ER) condition, we asked them to remember the stimuli associated with error feedback for subsequent recall. We coded the paradigm with the Matlab Psychophysics Toolbox extensions^16^. Each of the NR, RR, and ER conditions were separated into 20 blocks of 10 trials each. During an initial *choice phase* in each block, participants were instructed to select 10 cards sequentially by trial and error from 12 decks presented on the computer screen, with the goal of maximizing their rewards (Fig. 1, top row). For the RR and ER conditions only, the choice phase in each block was followed by a *recall phase*, during which subjects were required to recall the decks corresponding to the feedback type as indicated by the condition instructions (Fig. 1, bottom). At the beginning of the RR and ER conditions subjects were given instructions to remember either the rewarded decks or the decks followed by error-feedback, respectively, for later report in the recall phase. No recall instructions were provided in the NR condition. Participants did one block of practice trials (including both phases for the RR and ER conditions) prior to performing the task in each memory condition. See supplementary online methods for further details of the paradigm.

#### Data acquisition

The EEG was recorded from 53 electrode locations, 23 frontal, 9 central, 16 posterior, 2 mastoid, and 3 ocular channels using BrainVision Recorder software (Brainproducts, Munich, Germany). Electrodes were arranged according to the standard 10–20 layout^17^ and were referenced online to the average voltage across the channels. Vertical and horizontal ocular movements were recorded by an electrode placed under the right eye (re-referenced offline to FP2), and two on the outer canthi of the right and left eyes (re-referenced offline to each other) respectively. Electrode impedances were kept under 20 kΩ. Data were sampled at 500 Hz and high pass filtered by the amplifiers at 0.017 Hz.

### Data analysis

#### Pre-processing

Data pre-processing was performed in BrainVision Analyzer 2. A band-pass filter (0.1–100 Hz) was applied to the EEG data and epochs of EEG activity were selected from 1 s before to 1 s after the onset of feedback stimuli. Data were subsequently re-referenced to the average mastoids. Ocular artefacts were corrected by an algorithm^18^ implemented in the Analyzer software. Feedback segments were baseline-corrected by subtracting, for each channel, subject, and trial, the average voltage values during the 100 ms prior to the feedback stimulus, from the subsequent voltages in the epoch. EEG artifacts were identified and rejected according to the following criteria: (1) any abrupt change of voltage greater than 35 µV from one time sample to the next, (2) any difference between the negative and positive peaks in a 200 ms interval that exceeded 150 µV, (3) any activity that was consistently smaller than 0.5 µV in a 100 ms interval. Segments meeting these criteria were rejected for all channels. Nearest neighborhood channel interpolation (Hjorth algorithm embedded in BrainVision analyzer) was used for channels that caused more than a 15% trial rejection rate for each condition. After interpolation, 7% of trials were rejected on average across all recordings for all subjects. Data were exported to MATLAB for the time–frequency analyses. Topographical scalp maps were plotted with EEGLAB^19^.

#### Time–frequency analysis

To extract time–frequency information, the two-second epoch centered on the onset of feedback presentation was convolved with a seven-cycle complex Morlet wavelet that was linearly scaled based on the frequency range of 1–100 Hz. The time–frequency power was extracted relative to a 100 ms baseline before the feedback on each trial as 10 * log10 (trial power/average baseline power). Raw beta power values for every subject and condition were calculated as the average power in a the frequency of interest (20–30 Hz) for the first 500 ms following the presentation of feedback^15,20^. Here we included a larger time window (as opposed to 250–450 ms) to capture variability across single trial data. Then, to reduce the spatial dimensionality of the data, principal component analysis (PCA) was applied to extract components that accounted for the greatest spatial covariance in the data. See supplementary online methods for details of the PCA.

#### Classification

We tested our hypotheses about beta using multivariate pattern analysis, a common method where a model (classifier) is trained to distinguish between patterns of neural activity associated with two or more brain states^21^. Our hypothesis predicts a role for beta in communication across areas, particularly between frontal and posterior cortical sites; therefore we selected the 2 spatial PCA components that each explained greater than 10% of the total variance in beta power as features for the classification analysis (see supplementary online methods). This threshold was set to ensure that we included more than only the frontal component of beta power while limiting the components to only those that significantly contributed to the total variance. We trained the classifier on these 2 components of beta power elicited by reward and error feedback and then tested our predictions using this model. To be specific, we performed the following three steps to test our hypothesis that beta power is sensitive to task-related information irrespective of valence. First, we tested whether we could train a classifier on post-feedback beta power in the NR and RR conditions (which we expected would elicit similar patterns of beta power) to discriminate trials with reward feedback, which were task-relevant in these conditions (*Class TR*), from trials with error feedback, which were task-irrelevant in these conditions (*Class TI*). This analysis verifies whether beta power discriminates between reward and error trials in a typical reinforcement learning task, both when there is no secondary task (NR condition) and when rewards signal the importance of the preceding stimulus in relation to an upcoming recall task (RR condition). Second, we tested if this model would classify trials with reward feedback and trials with error feedback in the ER condition as *Class TI* and *Class TR*, respectively, in essence reversing the classes for reward and error trials as identified in the NR and RR conditions. Successful performance of the model on this test—which was trained on the NR and RR conditions but tested on the ER condition—indicates that the model is sensitive to task-related information (here what participants are told to remember) rather than to the valence of the feedback (reward vs. error). Third, we tested if this model could also classify trials in which the stimuli were later recalled versus trials in which the stimuli were not recalled into *Class TR* and *Class TI,* respectively, regardless of feedback valence and condition. Successful performance on this test for a model that was trained only to discriminate reward trials from error trials would indicate that post-feedback beta power predicts whether the preceding stimulus was later recalled, irrespective of whether the feedback indicated an error or a reward.

Note that, crucially, the model was trained only on trials in the NR and RR conditions, in which the reward feedback was task-relevant. Once trained, this model was then tested on the independent trials of the ER condition to verify whether it would classify beta power elicited by error feedback as task-relevant when the role of the feedback was reversed. Last, we tested the same model on post-feedback beta power irrespective of the valence of the feedback and of the recall condition, in order to verify whether it would classify the recalled stimuli as being more task-relevant than the non-recalled stimuli. See supplementary online methods for details of training and testing the model.

#### Behavior

Reaction times for all trials in the choice task (200 trials minus an average of 3 missed trials per subject) were averaged and compared using a repeated-measures ANOVA with the NR, RR, and ER conditions as within-subject factors. For the recall task, the reaction times for all recalled responses (on average 76 trials per subject) were averaged and compared using a repeated measures ANOVA with recall conditions (RR, ER) and recall accuracy (correctly recalled, incorrectly recalled) as within-subjects factors. Post-hoc paired t-tests were used where applicable. Statistics on variables with more than two levels were corrected using the Greenhouse–Geisser correction for sphericity.

## Results

### Behavioral analysis

**Figure 2:**
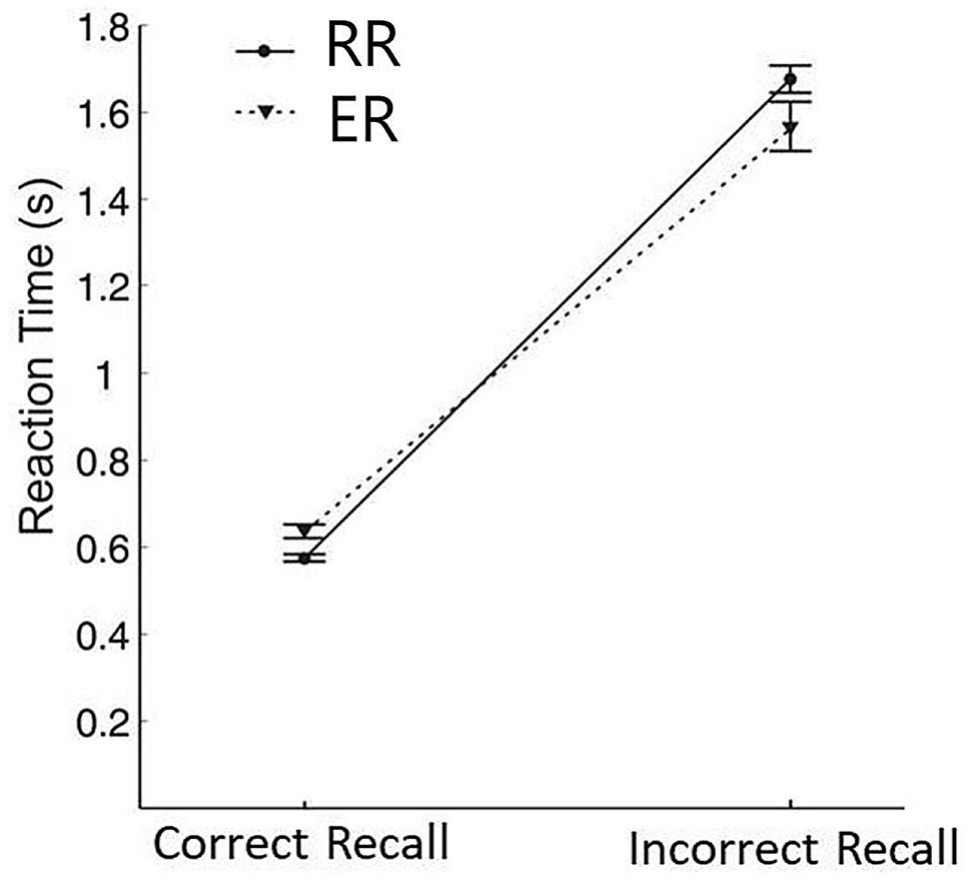
Reaction times during the recall phase. Correct and incorrect recalls in the Reward Recall (RR) and Error Recall (ER) conditions are illustrated. Error bars indicate standard errors.

Average reaction times across subjects during the choice phase were not significantly different across conditions (*p* > 0.05) (491 ± 160 ms in the NR condition, 533 ± 145 ms in the RR condition, and 531 ± 161 ms in the ER condition) suggesting that the memory instructions for the recall phase in the two recall conditions did not affect choice performance. A 2 × 2 ANOVA on reaction time with recall condition (RR, ER) and recall accuracy (correctly recalled, incorrectly recalled) revealed a significant main effect of recall accuracy (*F*(1,29) = 954.2, *p* < 0.001) and a significant interaction between accuracy and memory condition (*F*(1,29) = 19.7, *p* < 0.001) (Fig. 2). Post-hoc paired t-tests revealed that in the RR condition subjects were significantly faster when correctly reporting the decks associated with reward-feedback (571 ± 44 ms) compared to incorrectly reporting decks that were not associated with reward-feedback (1674 ± 179 ms) (*t*(29) = −40.5, *p* < 0.001). Likewise, in the ER condition participants were significantly faster when correctly reporting the decks associated with error-feedback (631 ± 88 ms) compared to incorrectly reporting the decks that were not associated with error-feedback (1558 ± 314 ms) (*t*(29) = −19.7, *p* < 0.001). Further, correct recalls were significantly faster in the RR condition compared to the ER condition (*t*(29) = −5.2, *p* < 0.001) and incorrect recalls were significantly slower in the RR condition compared to the ER condition (*t*(29) = 2.5, *p* = 0.019).

Recall accuracy was calculated as the percentage of correct recalls in each block averaged across the 20 blocks for the RR and ER conditions separately. Average recall accuracy was slightly but significantly higher in the RR condition (95.2 ± 4%) compared to the ER condition (90.7 ± 9%), (*t*(29) = 3.3, *p* = 0.003), indicating that participants were more accurate in recalling decks associated with reward-feedback compared to decks associated with error-feedback. We thus tested the model on recalled versus not recalled stimuli separately for the RR and ER conditions to account for the difference in accuracy across the conditions. Note that in order to equalize the task difficulty across the reinforcement learning (choice phase) and working memory (recall phase) tasks, the deck locations remained constant on the screen throughout both phases in each block, which possibly explains the relatively high recall accuracy in both conditions.

### Beta power

**Figure 3:**
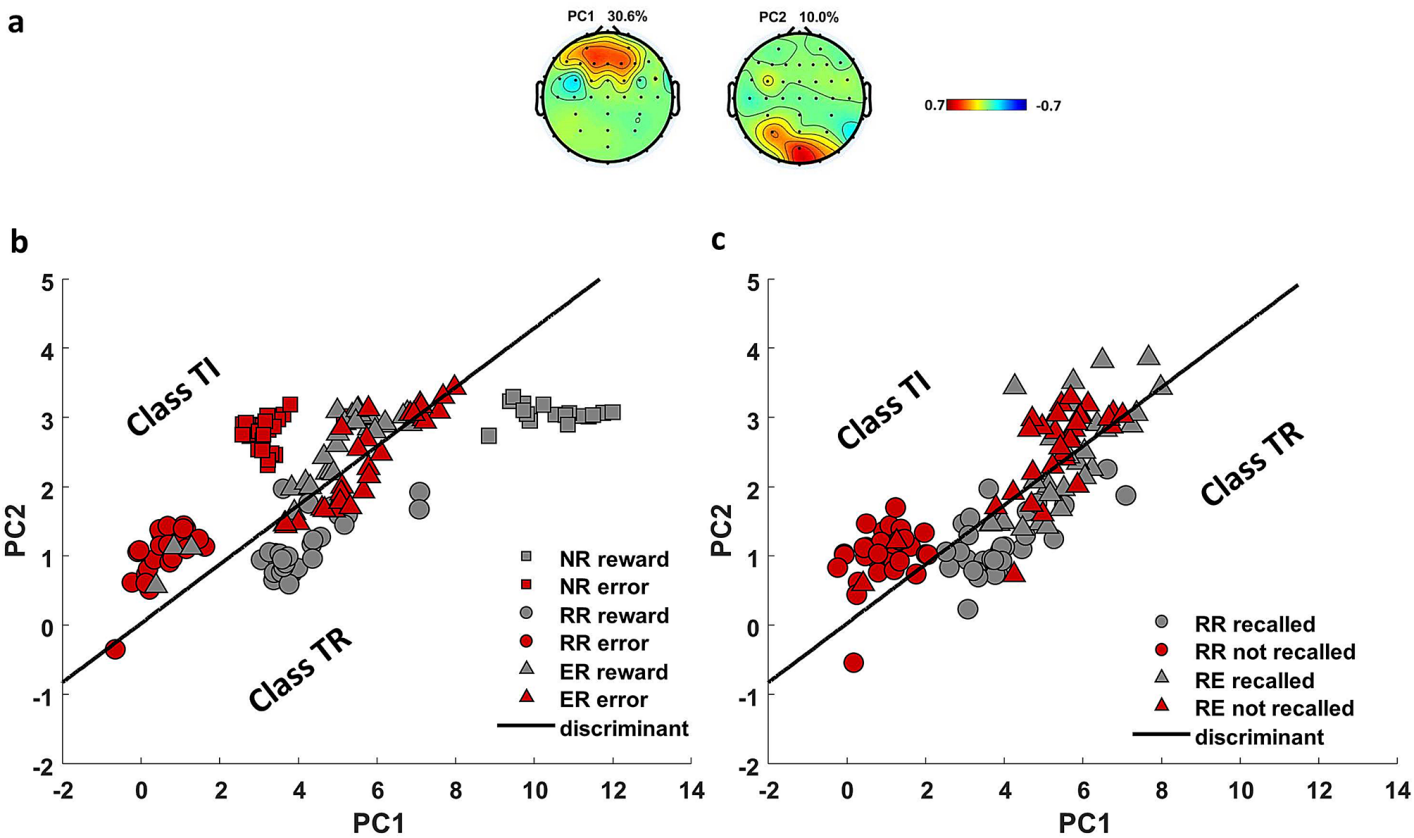
Discrimination of post-feedback beta power according to task demands. (**a**) Principal components derived from single-trial beta power in all conditions (RR, ER, and NR) that explained at least 10% of the variance. (**b**) Discriminant line represents the model that was trained by beta elicited to reward and error feedback in the NR (square) and RR (circle) conditions to classify rewards (gray) into task-relevant class (Class TR) and errors (red) into task-irrelevant class (Class TI). In the ER condition (triangle), this model could successfully discriminate errors (gray-Class TR) from rewards (red-Class TI), therefore reversing the assignment of reward and error trials, (**c**) Same discriminant model in (**b**) classified post-feedback beta associated to stimuli that were recalled (gray) versus those that were not recalled (red) into Class TR and Class TI respectively, regardless of valence and memory condition.

We selected the 2 spatial PCA components (frontal and occipital) that each explained greater than 10% of the total variance in beta power as features for the classification analysis (Fig. 3a; see “Materials and methods” section). We investigated whether beta power, as represented in a 2-dimensional space described by these two components, provides sufficient information to classify correctly the task-relevant (*Class TR*) versus task-irrelevant (*Class TI*) stimuli. When we trained the classifier on post-feedback beta power associated with the NR and RR conditions to discriminate the reward trials (*Class TR*) from the error trials (*Class TI*) (see Methods for details), the classification accuracy was 98% (*z*_*perm*_ = 10.5, *p*_*perm*_ < 0.01), which confirms that beta power differentiates between these stimulus conditions (Fig. 3b), as expected. Then we tested the model on post-feedback beta power associated with the ER condition to see whether the classifier is sensitive to task-related information as opposed to valence per se. To be specific, if the classifier is sensitive to task-related information then in the ER condition it should classify rewards as belonging to *Class TI* and errors as belonging to *Class TR*, i.e., the reverse classification of the RR condition. Consistent with this prediction, rewards and errors were classified as *Class TI* and *Class TR*, respectively, in 80% of the observations (*z*_*perm*_ = 4.8, *p*_*perm*_ < 0.01). This indicates that a model trained to distinguish reward versus error trials in one data set (the RR condition) can correctly classify trials in a different data set (the ER condition) according to their task relevance (i.e., what participants are instructed to remember) as opposed to their valence (reward vs. error) (Fig. 3b; Table 1, ER).

**Table 1:** Confusion matrix showing the number of correct and incorrect classifications by the model in each condition.

	ER (60 observations)		Recall-RR (60 observations)		Recall-ER (60 observations)	
	Reward	Error	Recalled	Not-recalled	Recalled	Not-recalled
Class *TR*	2	*20*	*27*	2	*18*	5
Class *TI*	*28*	10	3	*28*	12	*25*

Number of correct classifications in each column is presented in italics.

We thus set out to test the final prediction: that beta oscillations reflect the maintenance of information that supports task performance, which in the present case entails recalling the target stimulus (stimuli associated with reward in the RR condition and stimuli associated with errors in the ER condition) in the recall phase. Therefore, we predicted that stimuli that were subsequently recalled should evidence beta-related encoding during the choice phase that is similar to Class TR, irrespective of whether the recall was correct or incorrect. To test this, we averaged post-feedback beta power components according to whether the stimuli associated with feedback on that trial were recalled or not in the recall phase, separately for the RR and ER conditions and regardless of whether the recalls were correct or incorrect. Then, we tested whether the model that was trained based on feedback valence could also distinguish recalled versus not-recalled stimuli. In line with this prediction, when collapsing across the RR and ER conditions the discriminant model classified the recalled stimuli as *Class TR* and the not-recalled stimuli as *Class TI* with 82% accuracy (*z*_*perm*_ = 7.1, *p*_*perm*_ < 0.01) (Fig. 3c; Table 1). The accuracy was 92% in the RR condition and 72% in the ER conditions separately. The high accuracy of the model in the RR condition is not surprising as the overlap of rewarded stimuli (on which the model was trained) and recalled stimuli (on which the model was tested) was high. However, the high accuracy of the model in the ER condition, in which stimuli associated with error feedback were recalled, supports our hypothesis that beta power is not specific to rewards but rather reflects the task requirements. In other words, this test demonstrates that post-feedback beta power distinguishes between trials in which stimuli were recalled versus trials in which stimuli were not recalled, regardless of feedback valence and memory condition.

Post-feedback beta power averaged across feedback type was not correlated with subjects’ overall recall accuracy in either of the memory conditions (*p* > 0.05), perhaps because of a ceiling effect in accuracy levels (see above).

## Discussion

Previous studies have observed an increase in beta power over frontal areas of the scalp around 250–450 ms following the delivery of reward-related feedback stimuli^10,11,22^. Current proposals associate this oscillatory activity with learning from rewards^13^ through an interplay between neurocognitive processes related to motivation, attention and memory^14^. Previously, we localized the source of this signal to DLPFC and proposed that it relates to the maintenance and transfer of recently reinforced stimulus–response rules to brain areas that are responsible for task execution, thereby facilitating behavioral adaptation on subsequent trials^15^. On this view, reward feedback boosts the active representation (i.e., memory) of the immediately preceding action sequence. In contrast, error feedback does not elicit as much frontal beta power because error-related information in these tasks is less helpful for improving task performance. This view also suggests that beta power over posterior regions may reflect the transfer of that information to the neural areas responsible for task execution.

Here, we provide evidence for this proposal by showing that reward-related beta power is not specific to reward feedback per se but rather depends on the informative value of the feedback stimuli for task performance. In a control condition, subjects were asked to perform a relatively typical reinforcement learning card choice task (NR), and in two other conditions, the task demands were modified by asking subjects to remember choices that were followed by either reward (RR) or error (ER) feedback for later recall. In particular, the first two principal components of beta power successfully discriminated reward feedback (*Class TR*) from error feedback (*Class TI*) in the NR condition (Fig. 3a,b). This is consistent with previous studies that found higher frontal beta power elicited to reward compared to error feedback^9–11,22^. Further, although this pattern was unchanged by the instruction to remember rewards (Fig. 3b-RR), it was reversed by the instruction to remember errors, such that the model classified rewards to *Class TI* and errors to *Class TR* in the ER condition (Fig. 3b-ER). Finally, beta elicited to feedback following recalled stimuli was classified into *Class TR* whereas beta elicited to feedback following stimuli that were not recalled was classified into *Class TI*, irrespective of whether the feedback in fact indicated rewards or errors (Fig. 3c). This indicates that the beta signal following the feedback is associated with stimulus recall as per the task instructions, providing evidence for a broader function of beta oscillations beyond reward processing in particular.

We interpret these results in the context of evidence from non-human primate studies suggesting that beta oscillations in the PFC support the active maintenance of task representations by sending “top-down” control signals that regulate the function of other brain areas^23^. Local field potentials recorded from the principle sulci of monkeys, which are a homolog of human DLPFC^24^, show that beta oscillations reflect the activity of task-specific neural ensembles^25^, and that DLPFC neurons synchronize with striatal and hippocampal neurons when learning novel stimulus categories and associations^26–28^. Recent primate work also suggests that frontal beta oscillations track attentional effort, a process associated with maintaining good task performance despite fatigue^29^.

Human work paints a similar picture as work in animals. For example, joint analysis of EEG and functional magnetic resonance imaging in a gambling task has revealed an association between beta power and the BOLD response in the striatum, hippocampus, and DLPFC^30^, similar to the network of regions implicated in monkeys^26,27^. Frontal beta power has also been seen to increase during the maintenance period of a working memory task when performance is rewarded compared to when it is not^31^. Furthermore, similar to the animal work relating beta power to effort, evidence also indicates that frontal beta power in humans increases with memory load^32^, and that the theta-to-beta (low-gamma) frequency ratio is associated with working memory load^33^ and working memory capacity^34^. Interestingly, a recent study showed that post-feedback beta power difference between positive and negative feedback is attenuated by increasing the delay between the time of response and feedback presentation^35^, supporting the hypothesis that beta plays a role in maintaining and communicating information about preceding actions.

The 2-component classifier that we tested in the current study suggests that both frontal and posterior beta may be critical for this function. In line with the non-human animal literature, where the interaction between the PFC and hippocampus is found to be driven in part by synchrony in beta oscillations across the regions^27,36^, we speculate that in the current task, frontal post-feedback beta oscillations reflect a boost in the currently online memory of the preceding deck stimulus that facilitates the transfer of this information to hippocampal regions. By contrast, the posterior beta component may reflect the role of the occipital cortex in working memory processes^37,38^. This possibility is supported by evidence that occipital cortex activation is sensitive to task-related features of objects in visual working memory^39^ and that synchrony across occipital and frontal cortex predicts performance in visual working memory processes^40^. Further, beta oscillations could underlie a top-down mechanism of neural communication that facilitates encoding of stimulus properties across different regions^41,42^. Given the visual content of our paradigm, future research can verify our hypothesis about the posterior component of beta. Taken together with our previous findings^15^, this points to a role for beta in facilitating communication between the PFC and other brain regions according to task demands^43^.

In summary, we propose that frontal beta power reflects a neurocognitive mechanism mediated by the DLPFC that (1) signals the need for activating the memory of recent actions based on the desired task performance, and (2) facilitates the communication of this information to other brain regions for future task execution. In conventional reinforcement learning paradigms, such as in the NR condition here, beta power elicited by feedback discriminates reward and error stimuli (Fig. 3a,b) because rewards convey relatively more information for optimizing task performance. However, this process can be reversed by explicit memory instructions that increase the utility of error feedback relative to reward feedback.

## Supplementary information

Supplementary Information.
