# Supplementary material for: Beta oscillations following performance feedback predict subsequent recall of task-relevant information
Source: Sci Rep. 2020 Sep 15;10:15114. doi: 10.1038/s41598-020-72128-x (PMC7493892; doi:10.1038/s41598-020-72128-x)
Supplement: Supplementary file 1 — Supplementary Information. [file 41598_2020_72128_MOESM1_ESM.docx]

**Beta oscillations following performance feedback predict subsequent recall of task-relevant information**

**Supplementary Methods**

Azadeh HajiHosseini^a,^^[[1]](#footnote-1)*^, Cendri A. Hutcherson^b,c^, and Clay B. Holroyd^a,d^

aDepartment of Psychology

University of Victoria

P. O. Box 1700 STN CSC

Victoria, British Columbia

Canada, V8W 2Y2

bDepartment of Psychology

University of Toronto Scarborough

1265 Military Trail

Toronto, Ontario

Canada, M1C 1A4

chutcherson@utoronto.ca

^c^Department of Marketing

Rotman School of Management, University of Toronto

105 St George Street

Toronto, Ontario

Canada, M5S 3E6

^d^ Department of Experimental Psychology

Faculty of Psychology and Educational Sciences

Ghent University

Henri Dunantlaan 2

9000 Ghent, Belgium

clay.holroyd@ugent.be

Corresponding author: Azadeh HajiHosseini

Email address: hajihosseini@gmail.com

Declarations of interest: non

**Supplementary methods**

**Task:** At the start of each choice phase trial 12 fractal images (arranged across 3 rows and 4 columns; 19 cm×26.5 cm) were presented on a black screen (Figure 1, top row, far left). The images were described to subjects as representing 12 decks of cards, and maintained their locations throughout a block of ten of trials. Participants were instructed to use a left mouse button click to select one deck with the goal of maximizing their rewards. Five hundred milliseconds following the response, the selected card was shown in the center of the screen (6^o^ of visual angle) and remained on screen for 500 ms (Figure 1, top row, middle left). Then the feedback appeared as a cartoon fruit image on a gray background for 1000 ms, described to subjects as appearing on the back of the card (Figure 1, top row, middle right). The feedback stimuli consisted of an apple or orange indicating 5 cents (reward-feedback) or 0 cents (error-feedback), counterbalanced across participants, and a banana indicating 1 cent (“neutral feedback”) that was used to guarantee that the RR and ER conditions required performing different tasks (see below). The neutral condition had a low number of trials and was not meant to be utilized for data analysis, therefore the neutral feedback stimulus was not counterbalanced. For responses that exceeded 1.5 s, the trial was terminated and the next trial commenced; participants were not informed of this specific deadline but were instructed to respond fast enough to avoid misses. Feedback presentation was followed by a 1000 ms inter-trial interval (Figure 1, top row, far right). Participants could select a card from each deck only once; each selected deck was marked with a gray square in the corner of the image, indicating that it was unavailable for selection (Figure 1, top row, far left).

In the RR and ER conditions, in addition to the instructions to try to find as many rewards as possible, participants were also asked to remember which decks were associated with reward- or error-feedback, respectively. During the subsequent recall phase participants had 6 s to prepare to report the target decks, as indicated by the word “Remember…” presented in white font on a black screen (Figure 1, bottom row, far left). A white fixation cross was then presented in the center of the screen for 500 ms (Figure 1, bottom row, middle left) followed by the image of the 12 decks presented in the same locations as the choice phase (Figure 1, bottom row, middle right). Participants were instructed to click on the target decks according to the trial outcomes in the preceding choice phase. The recall phase terminated when all targets (decks associated with reward-feedback in the RR condition and decks associated with error-feedback in the ER condition) were selected, or after 10 s, whichever came first. The number of correct responses, i.e. the number of times the target decks were selected, was then presented on the screen for 2 s (Figure 1, bottom row, far right). There were no memory instructions given in the NR condition and the order of performing the RR, ER, and NR conditions was counterbalanced across participants. Following the recall phase in the RR and ER conditions and following the choice phase in the NR condition, the end of the block was indicated by text on the screen, allowing subjects to continue to the next block at their own pace.

In each block, the 12 stimuli were randomly drawn from a set of 24 fractal images that were downloaded from The Fractal World Gallery at <http://www.enchgallery.com/>. Abstract images were adopted in order to minimize the types of memory strategies that participants might employ relative to stimuli with semantic content. Fractal images have been used previously for reinforcement learning^1^ and working memory^2^ task paradigms.

Unbeknownst to the participants, the outcomes during the choice phase were predetermined and independent of their behavior. More specifically, the number of reward and error outcomes on each block varied randomly between 2 and 6 so that their sum was always 8, and the number of neutral outcomes was fixed at 2. The two neutral outcomes on each block were included to ensure that remembering the decks associated with either reward-feedback or error-feedback were not redundant tasks; without the neutral feedback, participants could have successfully performed the task by remembering only the decks associated with reward-feedback or error-feedback in both RR and ER conditions irrespective of the task instructions. The number of reward and error trials across blocks was selected at random from a uniform distribution between 2 and 6, which ensured a comparable number of trials for the reward and error conditions across the 20 blocks. Therefore, across the 20 blocks of 10 trials, about 40% were associated with reward outcomes, 40% were associated with error outcomes, and 20% were associated with neutral outcomes, yielding approximately 80 reward trials and 80 error trials total. The monetary reward was calculated and paid to the participants at the end of each condition of the task (~13.20 CAD).

Upon completion of the experiment all participants answered a brief, 5-question questionnaire that asked them to rate the difficulty of the conditions and explain what, if any, strategies they used to perform the task.

**PCA:** PCA was applied to a covariance matrix that was computed over the averaged-over-time raw beta power values for each single trial for all conditions (NR-reward, NR-error, RR-reward, RR-error, ER-reward, ER-error), subjects and channels, yielding multiple components representing localized clusters of EEG activity in beta frequency that covary spatially across the scalp^3^. More specifically, the input to the PCA was a matrix containing averaged beta power in the 500-ms window for every trial across all blocks and conditions (excluding the trials that were rejected for artefacts) in each row and 48 channels in each column. The first 15 components that together accounted for 80% of the total variance were submitted to Varimax rotation. Single-trial beta power for components that explained at least 10% of the variance was obtained by taking the inner product of the factor loadings with the single-trial raw beta power values recorded for each channel. Single-trial component beta power was re-grouped for each condition and subject. The 10% threshold was set to ensure including non-frontal components of beta power but limit to those that have a significant contribution to total variance.

**Classification:** To build the model, we used linear discriminant analysis^4^ using *fitcdiscr* in MATLAB in order to train a classifier on post-feedback beta power in the NR and RR conditions. Component beta power was averaged across single trials for each condition for each subject. The input to train the classifier was a matrix composed of 120 rows representing one observation for reward and error (averaged across trials) in the NR and RR conditions for each subject, and 2 columns for components that each explained at least 10% of the variance (Figure 3a). The target vector included 1 for the reward condition and 0 for the error condition. We then used *predict* to obtain the accuracy of the classifier on the training set, the same 120×2 matrix, as a sanity check for the model by calculating the number of observations that were correctly classified: Every observation related to reward that was classified as 1 (*Class TR*) and every observation related to error that was classified as 0 (*Class TI*) were considered correct (completing the first step described in the methods section of the main text). We then used *predict* to test the model performance on classifying the rewards and errors in the ER condition. The test input was a matrix composed of 60 rows representing one observation for each of the reward and error conditions in the ER condition for each subject and 2 columns for the first two components. Every observation related to reward that was classified in *Class TI* and every observation related to error that was classified in *Class TR* were considered correct (completing the second step explained in the methods section). We then used *predict* to test the model performance on classifying the recalled and not-recalled stimuli irrespective of valence and condition. The test input was a matrix composed of 120 rows representing one observation for the recalled and non-recalled stimuli (averaged across trials) for RR and ER conditions for each subject and 2 columns for the first two components. Every observation related to recalled stimuli that was classified in *Class TR* and every observation related to not-recalled stimuli that was classified in *Class TI* were considered correct (completing the third step explained in the methods section). For all of these analyses, observed accuracy was calculated as the percentage of correct classifications among all observations. Then the analysis was repeated for 5000 permutations whereby the labels (“reward”/“error” or “recalled”/“not recalled”) were shuffled at random and the accuracy for each permutation, *accuracy_perm,* was determined as per above. The statistical significance of the observed accuracy was given by a *z_perm_* and a *p_perm_* value where *z_perm_* = (*accuracy_observed* – mean(*accuracy_perm_1:5000_*))/ std(*accuracy_perm_1:5000_*) and *p_perm_* <0.01 if *accuracy_observed* is greater than at least 99% of values in *accuracy_perm_1:5000_*.


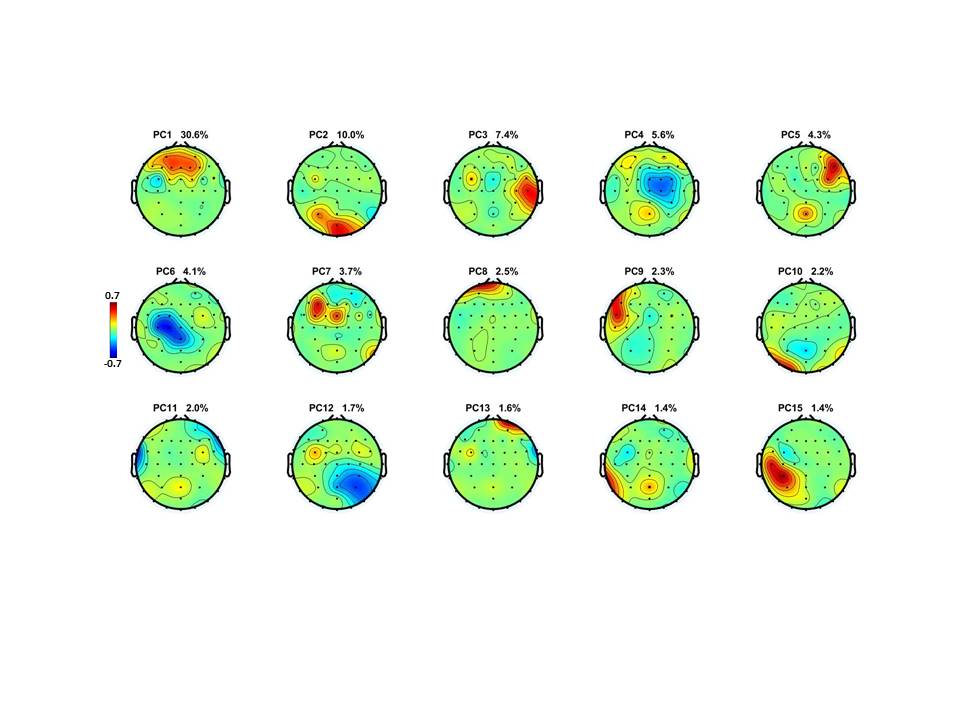


**Figure S1**: Varimax rotation was applied to 15 components that together explained 80 percent of the total variance following PCA on single trial beta power across all conditions and subjects. The components that explained at least 10% of the variance (PC1 and PC2) were used to build the classifier.

**References**

1. Gläscher, J., Daw, N., Dayan, P. & O’Doherty, J. P. States versus rewards: Dissociable neural prediction error signals underlying model-based and model-free reinforcement learning. *Neuron* **66**, 585–595 (2010).

2. Ragland, J. D. *et al.* Working memory for complex figures: an fMRI comparison of letter and fractal n-back tasks. *Neuropsychology* **16**, 370–379 (2002).

3. Spencer, K. M., Dien, J. & Donchin, E. Spatiotemporal analysis of the late ERP responses to deviant stimuli. *Psychophysiology* **38**, 343–358 (2001).

4. Donchin, E. & Hefﬂey, E. Multivariate analysis of event-related potential data: A tutorial review. *Multidisciplinary perspectives in event-related brain potential research* 555–572 (1978).

1. Current affiliation:

   Department of Psychology

   University of Toronto Scarborough

   1265 Military Trail

   Toronto, Ontario

   Canada, M1C 1A4

   azadeh.haji@utoronto.ca [↑](#footnote-ref-1)
